# Percutaneous Fixation for Traumatic Symphysis Pubis Disruption—Are the Results Superior Compared to Open Techniques? A Systematic Review and Meta-Analysis of Clinical and Biomechanical Outcomes

**DOI:** 10.3390/jcm12154988

**Published:** 2023-07-28

**Authors:** Dimitrios Kitridis, Konstantinos Tsikopoulos, Panagiotis Givissis, Byron Chalidis

**Affiliations:** 1 1st Orthopaedic Department, School of Medicine, Faculty of Health Science, Aristotle University of Thessaloniki, 54124 Thessaloniki, Greece; pgivissis@gmail.com (P.G.); byronchalidis@gmail.com (B.C.); 2 1st Department of Pharmacology, School of Medicine, Faculty of Health Science, Aristotle University of Thessaloniki, 54124 Thessaloniki, Greece; kostastsikop@gmail.com

**Keywords:** traumatic, pubic symphysis diastasis, pelvic fractures, percutaneous cannulated screw, minimally invasive, reconstruction plate, biomechanics

## Abstract

Introduction: Open reduction and reconstruction plate and screws fixation (RPSF) is considered the gold standard for the treatment of traumatic symphysis pubis diastasis (SPD). Percutaneous cannulated screw fixation (PCSF) has recently gained popularity as it may reduce operative time and morbidity. The current systematic review aims to compare the clinical and radiological outcomes of PCSF and RPSF in traumatic SPD and analyze the biomechanical effectiveness of PCSF. Material and Methods: The Medline, Scopus, and Cochrane databases were searched until February 2023. The primary outcomes were the incidence of implant failure and revision surgery and the amount of displacement of symphysis pubis. Secondary outcomes were the intraoperative blood loss, the scar length, the operative time, the wound infection, and the patients’ functional improvement. Results: Six clinical trial studies with a total of 184 patients and nine biomechanical studies were included. There was no significant difference between the two groups regarding the incidence of implant failure, the prevalence of revision surgery, and the amount of postoperative loss of reduction (*p* > 0.05 for all outcomes). The intraoperative blood loss (14.9 ± 4.2 mL for PCSF versus 162.7 ± 47.6 mL for PCSF, *p* < 0.001) and the incision length (1.7 ± 0.9 mL for PCSF versus 8 ± 1.4 mL for PCSF, *p* < 0.001) were significantly lower after PCSF. The mean operative time was 37 ± 19.1 min for PCSF and 68.9 ± 13.6 min for RPSF (*p* < 0.001). The infection rate was less frequent in the PCSF group (3% for PCSF versus 14.3% for RPSF, *p* = 0.01). One clinical trial reported better functional recovery after PCSF. In all biomechanical studies, the threshold for implant failure was beyond the applied forces corresponding to daily activities. Conclusions: PCSF for traumatic SPD is associated with less operative time, less blood loss, and a lower infection rate when compared to conventional plate techniques without increasing the incidence of postoperative fixation failure and revision surgery. Moreover, PCSF has been proven to be biomechanically sufficient for stabilization. Therefore, it should be considered an efficient and viable alternative for the reconstruction of SPD when closed reduction can be adequately achieved.

## 1. Introduction

The incidence rate of pelvic and acetabular fractures is increasing as a result of the increased occurrence of high-energy injuries caused by traffic accidents or falling from high places [1,2]. The mortality risk following pelvic fractures ranges from 5% to 20%, which is a remaining challenge in the field of orthopedic and trauma surgery [2]. The modified Tile classification has been widely used to describe the fracture patterns, allowing assessment of the stability of the pelvic ring [1]. Symphysis pubis diastasis (SPD) occurs in approximately 24% of pelvic fractures and may be associated with other pelvic ring injuries [1,2]. Simple SPD, named the “open book” lesion, is rotationally unstable and is characterized as type B1 according to the Tile classification [2]. Widening of the symphysis pubis (SP) greater than 25 mm implicates that the anterior sacroiliac ligaments are mostly damaged and is considered an indication for surgery [3], which may be performed either alone or simultaneously with posterior pelvic ring fixation according to the integrity of the posterior pelvis [4,5].

Open reduction and reconstruction plate and screws fixation (RPSF) via a Pfannenstiel approach is considered till now the standard treatment for unstable SP injury, providing relatively easy access to the anterior pelvic ring and a low incidence of incisional hernia [6]. However, the technique has several disadvantages. Although the anatomy of the anterior pelvis is well-described, the exposure of the symphysis pubis may cause significant blood loss, as well as neural and vascular injuries [7]. The lateral extension of the incision can damage the inguinal canal contents and lead to chronic pain disability [8]. Furthermore, wound problems, especially in obese and diabetic patients, and heterotopic bone formation may be encountered [9].

With the improvement of intraoperative imaging, several alternative less traumatic fixation methods have been recently introduced and gained popularity as they could reduce operative time and morbidity. Minimally invasive [10] or endoscopic plate fixation [11,12], percutaneous cannulated screw fixation (PCSF) [8,13], Endobutton technique for dynamic fixation [10], and tape suture fixation [14] promise not only adequate stability of the disrupted anterior pelvic ring but also smaller skin incisions, less soft tissue trauma, and minimal blood loss [2]. However, published reports of PCSF for SPD are quite rare, and the overall value and superiority of the technique remain unclear. The purpose of the current systematic review is to compare the outcomes and complication rates of PCSF and RPSF in traumatic SPD and present the available evidence regarding the biomechanical effectiveness and safety of different PCSF options.

## 2. Material and Methods

### 2.1. Search Strategy and Eligibility Criteria

The present review was performed in agreement with the Preferred Reporting Items for Systematic Reviews and Meta-Analyses (PRISMA) guidelines, albeit not a priori registered [15]. A systematic search of the Pubmed, Scopus, and Cochrane Central Register of Controlled Trials databases was performed until February 2023. The following search string was used: “(pubi*) AND (symphys*) AND (percutaneous OR minimally)”. No date limits or additional filters were utilized. The references of the included articles were further manually searched for additional studies. Two authors independently screened the relevant records for inclusion.

Articles were included if they met one of the following criteria:

1. The study reported clinical outcomes after the application of PCSF either alone or in comparison with RPSF for traumatic SPD in patients 16 years of age or older.
2. The study reported biomechanical or anatomical properties of PCSF in cadaveric or software simulation studies.

Articles were excluded if they met the following criteria:

1. The report was a conference abstract.
2. The study did not present clinical or biomechanical data, such as reviews and letters to the editor.
3. The study evaluated nontraumatic SPD.
4. The study was not written in the English language.

### 2.2. Data Extraction

Two authors independently reviewed and extracted data from the selected articles including studies’ (type of study, year, country) and patients’ characteristics, surgical interventions, outcomes, complications, and length of follow-up period. The primary outcomes of the study were the incidence of implant failure and revision surgery, as well as the amount of post-surgery displacement of the symphysis pubis as measured at the immediate postoperative and latest follow-up radiographs. Secondary outcomes were the intraoperative blood loss, the scar length, the operative time, the incidence of wound infection, and the patients’ functional outcomes. Information considering the biomechanical efficacy of the fixation techniques was based on the threshold values for implant failure and specifically whether the fixated symphysis could withstand the applied forces corresponding to daily activities of sitting, standing, and walking. Furthermore, data from anatomical studies were analyzed also regarding the distance of the screws’ trajectories from major structures and whether any injuries were reported.

### 2.3. Quality Assessment

For clinical case series studies, the Moga et al. [16] checklist was used (a score of 13–18 indicates high quality, 7–12 moderate, and 0–6 low quality). The Coleman et al. [17] score was applied for the quality assessment of clinical comparative studies (a scale of 0 to 100; a score of 100% is considered the perfect score and indicates high quality).

Regarding the biomechanical and anatomical studies, there is currently no validated quality appraisal tool. The assessment was based on a modified checklist developed by Dewan et al. [18]. This checklist is a combination of the relevant elements of the Critical Appraisal Skills Programme (CASP) tool and the Quality Appraisal for Cadaveric Studies (QUACS) scale [19,20].

### 2.4. Statistical Analysis

The meta-analysis was performed with the Review Manager software (RevMan Version 5.3, Copenhagen: The Nordic Cochrane Centre, The Cochrane Collaboration, 2014) using the effect size of standardized mean difference according to the inverse variance method and a random-effects model because of the anticipated heterogeneity across studies [21]. Only studies with direct comparisons were included in the meta-analysis. If a meta-analysis was not feasible, continuous data were pooled using weights according to each study’s sample size and compared using the two-tailed Student’s *t*-test assuming equal variances between the two groups. Categorical outcomes were compared using the chi-squared test. Microsoft Excel version 16 and IBM Statistical Package for Social Sciences (SPSS) software version 24 were used for the analyses. The level of significance was set at *p* < 0.05.

## 3. Results

**Figure 1:**
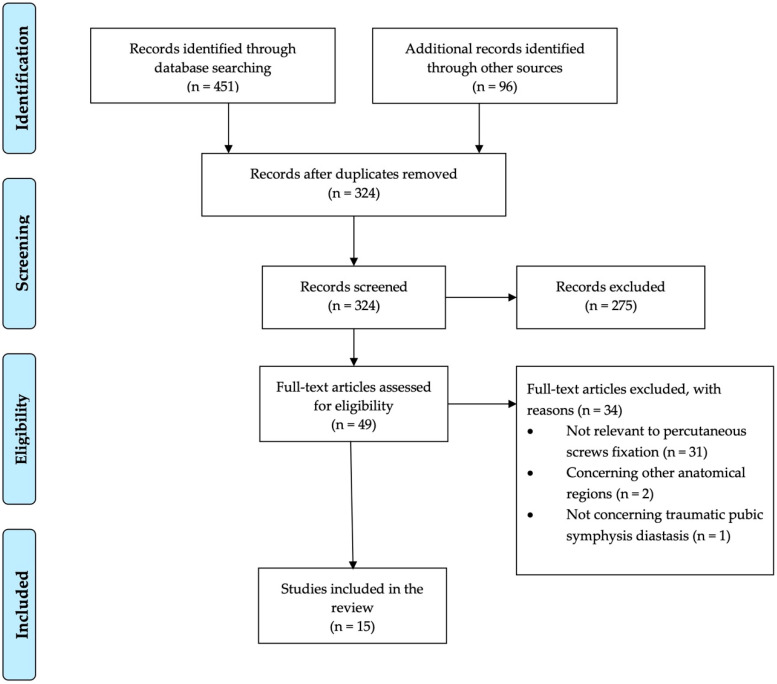
PRISMA flow diagram of the study.

The initial database and manual search identified 547 articles, of which 223 were duplicates and 275 were excluded using the titles and abstracts. Finally, 15 articles including six clinical and nine biomechanical studies fulfilled the eligibility criteria and were considered to be relevant for this review. The flow chart of the selection process is presented in Figure 1.

### 3.1. Characteristics of Clinical Studies

Six clinical studies described PCSF for traumatic SPD [2,3,7,22,23,24]. In more detail, one prospective randomized trial [22] and one retrospective non-randomized trial [2] compared PCSF with RPSF, one prospective randomized comparative trial compared PCSF with a TightRope (Arthrex, Naples, FL, USA) device combined with an external fixator [7], one retrospective case series study evaluated PCSF [23], and two retrospective case series studies evaluated computer-navigated PCSF [3,24]. These studies enrolled 219 patients in total, and the number of patients completing the follow-up assessments was 114 for PCSF and 70 for RPSF. The publication dates ranged between 2009 and 2022. Five studies were conducted in Asia [2,3,7,22,24], and one in the United States of America [23]. The clinical studies’ characteristics are presented in Table 1.

### 3.2. Percutaneous Fixation Technique

Regarding PCSF, closed reduction of the SPD was achieved by using two Schanz pins that were inserted into both iliac crests and large pointed reduction clamps [2,3,7,22]. Afterward, a K-wire was introduced between the pubic tubercle and the ipsilateral superior ramus and was forwarded to the contralateral superior ramus under fluoroscopy guidance. Then, a 6.5 or 7.3 mm short-threaded cannulated screw was inserted along the K-wire [2,3,7,22,23]. In the case of concomitant posterior pelvic disruption, posterior fixation was also performed with percutaneous sacroiliac screws. Mu et al. [3] and Chan et al. [24] used computer navigation and implanted a second screw to achieve improved stability if anatomy and execution were possible. In more detail, in cases of posterior pelvic disruption or multiple rami fractures, Mu et al. [3] inserted a second crossed screw from the base of the pubic tubercle to the superior part of the opposite side body of the pubis. Furthermore, Chen et al. [22] applied the PCSF technique in patients with vertical shear pelvic injuries, after correcting the vertical displacement with 10–12 kg supracondylar traction for several days.

### 3.3. Characteristics of Biomechanical and Anatomical Studies

Nine studies published from 2012 to 2022 investigated the biomechanical properties of PCSF [2,25,26,27,28,29,30,31,32]. Four studies utilized cadaveric specimens [25,26,27,28], two conducted finite elements analyses [2,29], two performed both cadaveric and finite element analyses [30,31], and one utilized composite pelvis models [32]. Five trials were conducted in Asia [2,27,29,30,31], two in Europe [25,26], and one in the United States of America [32]; one included authors from both Asia and the United States of America [28] (Table 2).

### 3.4. Quality Assessment

Two clinical studies provided level II evidence [7,22], one provided level III evidence [2], and three provided level IV evidence [3,23,24]. The comparative clinical trials were rated with a mean Coleman methodology score of 82.3% (range 63–93%) [2,7,22]. The case series studies by Eakin et al. [23] and Chan et al. [24] were of high quality (14 out of 18 points), and the study by Mu et al. [3] was of moderate quality (10 out of 18 points) according to the Moga score.

All biomechanical and anatomical studies utilized mechanical setup and parameters, which were representative of the in vivo biological conditions (Table 2). However, most studies did not explore repetitive loadings or tissue adaptation over time. The detailed critical appraisal of the biomechanical studies according to the checklist developed by Dewan et al. [18] is presented in Table 3.

### 3.5. Primary Outcomes

Implant failure was reported in 11 patients (9.6%) after PCSF and in 10 patients (14.3%) after RPSF. However, this difference did not reach statistical significance (*p* = 0.34). The incidence of revision surgery due to postoperative displacement and construct failure was similar in both groups as it was required in four patients in the PCSF group (3.5%) and in seven patients in the RPSF group (10%), (*p* = 0.07) (Table 4).

The mean difference between immediate postoperative and final follow-up SP width was 0.62 ± 1.33 mm and 1.17 ± 2.45 mm after PCSF and RPSF, respectively. However, this change was not statistically significant (*p* = 0.07).

### 3.6. Secondary Outcomes

**Figure 2:**
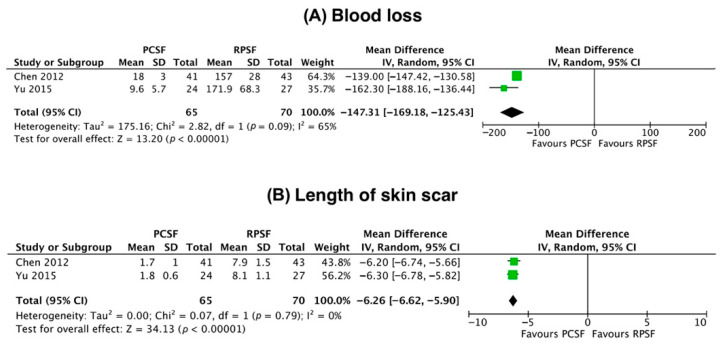
Forest plots demonstrating the mean difference of intraoperative blood loss (**A**) and length of skin scar (**B**) between percutaneous cannulated screw fixation (PCSF) reconstruction plate and screws fixation (RPSF). Chen et al. [15], 2012; Yu et al. [2], 2015.

The mean intraoperative blood loss for PCSF was 14.9 ± 4.2 mL, while for RPSF it was 162.7 ± 47.6 mL. The mean skin incision length was 1.7 ± 0.9 cm for PCSF and 8 ± 1.4 cm for RPSF. The meta-analysis demonstrated a statistically significant difference in favor of PCSF for both parameters (*p* < 0.001 for both outcomes, Figure 2).

The mean operative time was significantly shorter for PCSF than for RPSF (37 ± 19.1 versus 68.9 ± 13.6 min, respectively, *p* < 0.001). However, the duration of surgery was significantly increased after the application of computer navigation for PCSF (57 ± 12.5 minutes, *p* < 0.01, when compared to non-navigated PCSF).

Superficial infection was more frequent after RPSF rather than PCSF (10 patients, 14.3%, versus three patients, 3%, respectively, Table 4) (*p* < 0.01). All patients were treated conservatively with antibiotics and frequent wound dressing changes. No case of deep infection was reported.

Regarding functional outcomes, four studies reported a scoring system described by Majeed et al. [33], which utilized clinical information such as pain, sitting, sexual intercourse, walking, and working [2,7,22,24]. Two studies compared PCSF with RPSF [2,22], and one study compared PCSF with a TightRope technique [7]. Only the study by Chen et al. [22], which compared PCSF with RPSF, reported better functional recovery after PCSF (Table 5).

### 3.7. Biomechanical Outcomes

The research setups utilized axial loads imitating single- and dual-leg standing [2,25,26,32], as well as axial loads combined with rotational torque loads [29]. In all biomechanical studies, the threshold of failure was beyond the applied forces of daily activities and standing. In the comparative biomechanical studies, the cannulated screws provided comparable biomechanical properties to plate fixation [2,26,29,32]. Overall, the authors concluded that PCSF was biomechanically adequate to resist failure after surgery (Table 2).

Four anatomical studies assessed the accuracy and safety of screw positioning by determining the trajectories of the screws and the relevant distances from the surrounding major anatomical structures [27,28,30,31]. The studies did not report any inadvertent injury of neurovascular or soft tissue elements and consequently verified that fluoroscopy guidance may guarantee the accurate introduction of the screws.

## 4. Discussion

The present systematic review showed that PCSF is a successful alternative for the treatment of traumatic SPD. According to the available data, there is no significant difference between standard open and percutaneous fixation techniques regarding the incidence of implant failure, the prevalence of revision surgery, and postoperative SP displacement. Compared to RPSF, PCSF has been associated with shorter operative time, less intraoperative blood loss, and shorter skin incision length (*p* < 0.001 for all parameters). However, the functional outcomes and the infection rates were found not to differ significantly across the study groups.

Based on the available clinical studies, we noticed a low rate of implant failure after PCSF, which was similar to the traditional plate fixation method (10% and 14.3%, respectively, *p* = 0.38). Eakin et al. [23] reported only one case of loss of reduction after 10 weeks postoperatively among the 12 patients treated with PCSF due to SPD. Interestingly, there was no radiographic evidence of screw breakage. Both Chen et al. [22] and Yu et al. [2] found similar rates of implant failure between the PCSF and RPSF groups (*p* = 0.39 and 1, respectively). Moreover, Chen et al. [22] recommended earlier hardware removal following PCSF since they considered that screw fixation was biomechanically more stable than RPSF. They advocated screw removal after 10 months postoperatively, particularly in young female patients to facilitate uneventful childbirth.

According to the current systematic review, the mean operative time is shorter in PCSF when compared to open fixation techniques (*p* < 0.001). On the other hand, some minimally invasive techniques are quite demanding, and therefore the surgical time may be prolonged. Specifically, Feng et al. [7] reported that the Tightrope technique combined with external fixation lasted 48.5 ± 9.4 min. Similarly, Mu et al. [3] recorded a longer operative time in eight patients with SPD who were operated on with a percutaneous lag screw under a fluoroscopy-based Iso-C3D computerized navigation system (57 ± 12.5 min). However, due to the paucity of available data, more studies are necessary to establish the beneficial effect of computer navigation in PCSF.

Among the proposed benefits of a minimally invasive procedure are reduced blood loss and operative time. Chen et al. [22] reported that patients treated with PCSF exhibited less intraoperative blood loss (18 ± 3 mL for PCSF versus 157 ± 28 mL for RPSF, *p* < 0.01) and extensive exposure (mean skin incision length for PCSF 1.7 ± 1 cm versus 7.9 ± 1.5 cm for RPSF, *p* < 0.001) than those who received plate fixation. Similarly, Yu et al. [2] enrolled patients with isolated Tile type B1 injuries and showed that the blood loss and the length of the skin incision in the PCSF group were significantly smaller than those in the RPSF group (blood loss 9.6 ± 5.7 mL versus 171.9 ± 68.3 mL, skin incision length for PCSF 1.8 ± 0.6 cm versus 8.1 ± 1.1 cm, respectively, *p* < 0.001 for both outcomes). Regarding the parameter of functional improvement, only Chen et al. [22] reported better and quicker functional recovery in favor of PCSF. This can be explained by the limited tissue trauma and subsequent faster healing process. The iatrogenic injury caused by detachment of the distal insertion of the rectus abdominis muscle to obtain adequate exposure of the SP during RPSF may delay patients’ recovery and predispose to abdominal hernia formation [34].

In terms of fixation stability and potential postoperative displacement, the comparable results between the two techniques reflect the effectiveness of the PCSF, which has been also documented in biomechanical studies. Recent literature has shown that the forces across the disrupted symphysis pubis are transmitted through the plate during the rehabilitation phase [35]. The plate–screw construct represents an eccentric–extramedullary fixation of the SP, in contrast with the cannulated screws only. The latter option acts as an intramedullary device and carries the biomechanical benefit of decreasing the number of stresses transmitted by the implant. Therefore, the cannulated screws may result in a lower failure rate [36]. Cano-Luis et al. [25] compared the biomechanical properties of the intact symphysis pubis and SPD fixed with PCSF and found that there was no significant difference in the mean displacement after the application of an axial load of 300N (*p* > 0.7). The authors advocated that the cannulated screws could effectively resist rotational forces and offer adequate stability of the anterior pelvic ring. Gonzálvez et al. [26] performed a biomechanical study in fresh human pelvis specimens simulating an AO B1.1 injury that was fixed with two cannulated screws or a 6-hole non-locked plate. After axial load application of 300N, the cannulated screws fixation was associated with better stability and superior biomechanical behavior compared to plate fixation. 

Dual fixation has been also recommended to improve the strength of the construct and minimize the incidence of loss of reduction. Yao et al. [29], in a 3-dimensional finite element model of SPD (Tile type B1), observed that dual fixation of SPD with a superior and anterior plate (dual-plate) or crossed dual cannulated screws (cross-screw) offered better anterior and posterior pelvic stability than single superior plate or single cannulated screw constructs. However, the clinical implications of their study are yet to be determined. Yu et al. [2], in another finite element analysis study, found that PCSF and RPSF were equally adequate and effective for SP fixation as the maximum observed displacement of SP was 0.643 and 0.408 mm, respectively.

When considering the safety of the PCSF technique, the published studies did not report any injury to the major structures and consequently verified that fluoroscopy guidance may guarantee the accurate introduction of the screws. Sun et al. [27] in a cadaveric study measured the distance of the screw corridors from the nearby major structures. They reported a minimum distance between the entry point and the spermatic cord (fallopian arch in the female) of 9 mm. Similarly, Yu et al. [30] found that the mean distance between the pubic tubercle and the round ligament of the uterus or the spermatic cord was 4.408 ± 0.304 mm, and 5.196 ± 0.251 mm, respectively. On the subject of the appropriate screw entry points, Liu et al. [31] provided the anatomical basis for implant insertion using a finite elements model as well as 16 cadaveric specimens. They observed that regardless of patients’ gender, the introduction of the screws at approximately 5 mm above the anterior inferior iliac spine and 10 mm outside the midline of the symphysis pubis was a safe procedure.

Although PCSF is applied mostly in Tile type B1 injuries, its indications may be further expanded to SPD injuries combined with vertical instability of the sacroiliac joint [3,22]. Chen et al. [22] corrected the vertical pelvis displacement by application of 10–12 kg supracondylar traction for several days before surgery. Moreover, in the presence of SPD along with pubic rami fractures, another percutaneously inserted screw towards the broken ramus may be necessary to stabilize the anterior ring. [3,22]. However, percutaneous fixation should be applied with caution or even avoided in case of regional infection, bladder injury, or incarceration during closed SPD reduction and open or comminuted fractures [3,22]. Obesity is also considered a relative contraindication for PCSF, as screw insertion may be hindered by the circumference of the thighs [3]. A figure-of-four position of the contralateral lower limb with the manual pressure of the contralateral proximal thigh are proposed to minimize the amount of soft tissue blocking the trajectory and to increase the amount of working space for the surgeon [24].

### Limitations

The current study has some limitations. From a methodology point of view, all the published data were not comparative, so it was not feasible to perform a meta-analysis with direct comparisons of all outcomes. Moreover, three clinical studies were retrospective and therefore might introduce selection or recall bias. From biomechanical point of view, all the relevant studies simulated a Tile B1 pelvic injury. However, in clinical practice, most of the patients have a combination of SPD with other anterior and/or posterior pelvic injuries. In addition, in a highly urgent and demanding emergency situation, the application of a percutaneous minimal invasive technique might be not as good as it may be in cadaveric studies or controlled operative settings. This can influence the outcomes and the effectiveness of PCSF. 

## 5. Conclusions

PCSF for traumatic SPD has all the advantages of a minimally invasive procedure, including less blood loss, minimal morbidity, and rapid recovery. The technique has been proven biomechanically effective to offer stability to the anterior pelvic ring and should be considered a reliable alternative to conventional plate fixation. Nevertheless, it is a challenging and demanding procedure with a long learning curve and higher intra-operative radiation exposure, and its application should be utilized in specific injury patterns.

## Figures and Tables

**Table 1:** Characteristics of the included clinical studies.

Study	Technique	N	Mean Age (yr)	Males/Females	Mean Follow-Up (mo)
Chan et al. [24], 2022	Single or dual 6.5 mm PCSF	13	57.9 (24–95)	8/7	At least 6
Chen et al. [22], 2012	A. Single 7.3 mm PCSF	41	32 ± 9	29/12	21 (18–26)
	B. RPSF	43	26 ± 11	33/9	
Eakin et al. [23], 2022	Single or dual 5.5, 6.5, or 7.3 mm PCSF	12	44 (16–76)	10/2	15 (10.7–27.7)
Feng et al. [7], 2016	A. Single 6.5 mm PCSF	16	33.2 ± 5.8	11/5	15 (12–20)
	B. Tightrope and ex-fix	10	32.5 ± 6.2	7/3	
Mu et al. [3], 2016	Single or dual crossed 7.3 mm PCSF, computer navigation	8	40.9 ± 17.9	6/2	16.1 ± 2.5
Yu et al. [2], 2015	A. Single 6.5 mm PCSF	24	33.4 ± 9.1	15/9	29.4 ± 8.8
	B. RPSF	27	34.8 ± 11.7	19/8	

PCSF: percutaneous cannulated screw fixation; RPSF: reconstruction plate and screws fixation.

**Table 2:** Characteristics of the included biomechanical and anatomical studies.

Study	Fracture Simulation	Implants	Testing Method	Outcomes	Specimens	Results/Conclusions
Cano-Luis et al. [25], 2012	Tile B1	Dual 6.5 mm PCSF	Axial load 300N (dual-leg standing)	Displacement of pubic symphysis	10 cadavers	PCSF biomechanically sufficient
Gonzálvez et al. [26], 2016	Tile B1	A. Dual 6.5 mm PCSF B. 3.5 mm superior RPSF	Axial load 300N (dual-leg standing)	Displacement of pubic symphysis	9 cadavers	PCSF more stable than RPSF
Liu et al. [31], 2022	Intact pelvis	Single 7.5 mm PCSF	Determination of the optimal insertion point and safe channels of screws	Screws diameter and length, distance between screw and anterior inferior iliac spine, coronal, sagittal, and horizontal plane angles	A. 3D finite element model analysis B. 16 cadavers	Screw length 47.0 ± 2.0 mm (M) and 39.8 ± 3.9 mm (F), diameter 7.1 ± 0.4 mm (M) and 6.1 ± 0.4 mm (F), distance between screw and AIIS 5.5 ± 0.5 mm (M) and 5.6 ± 0.7 mm (F), angle of coronal plane 55.9° ± 1.3° (M) and 50.7° ± 1.5° (F), angle of sagittal plane 26.7° ± 0.5° (M) and 24.1° ± 0.9° (F), and angle of horizontal plane 64.8° ± 0.6° (M) and 58.8° ± 0.8° (F). Safe screw insertion 5 mm above AIIS, and 10 mm outside the midline of the symphysis pubis.
O’Neill et al. [32], 2022	Tile C1	A. 6.5 mm 4-hole plate and 6.5 mm S1 transsacral screw B. Single 6.5 mm cannulated stainless steel screw and 6.5 mm S1 transsacral screw	7 mm vertical compressive displacement through the sacrum at a rate of 2 mm/s (single-leg standing)	Displacement and rotation in 3 dimensions at the sacroiliac joint and pubic symphysis, stiffness at maximum stroke distance	A. 4 B. 4	There was no significant difference in net displacement at both sacroiliac joint and pubic symphysis. There was significantly less rotation but more dis-placement in the screw group in the Z-axis. The screw group showed increased stiffness compared with the plate group.
Sun et al. [27], 2016	Intact pelvis	Dual crossed 6.5 mm PCSF	Optimization of the secure trajectory of crossed screws using computer navigation	Trajectory, mean screw length, distance from surrounding major structures	15 cadavers	Mean screw length 7.0 ± 4.2 and 7.1 ± 3.8 cm. Minimum distance between entry point and spermatic cord (fallopian arch in the female) was 9 mm. All screw corridors were intact. Computer navigation is reliable for PCSF. The trajectories of crossed screws are reliable and safe.
Xu et al. [28], 2016	Intact pelvis	A. Single 7.3 mm PCSF/fluoroscopy B. Single 7.3 mm PCSF/2D fluoroscopic navigation C. Single 7.3 mm PCSF/3D fluoroscopic navigation	Determination of the accuracy of screw position, instrumentation time, and fluoroscopic time	Malposition rate, mean instrumentation time, mean fluoroscopic time	6 cadavers	3D fluoroscopic navigation showed a higher accuracy rate in positioning and a shorter instrumentation time. The fluoroscopic time was the shortest in 2D fluoroscopic navigation.
Yao et al. [29], 2015	Tile B1	A. Superior RPSF B. Superior and anterior RPSF C. Single 7.3 mm PCSF D. Dual crossed PCSF (7.3 and 6.5 mm) E. Dual parallel PCSF (7.3 and 6.5 mm)	A. Axial load 500 N (dual-leg standing) B. Axial load 750 N (single-leg standing) C. Axial load 500 N, torque 7 Nm (rotation)	Construct stiffness, incremental micromotion of anterior and posterior pelvic ring, incremental rotational angle of anterior pelvic ring	3D finite element model analysis	Dual crossed PCSF and dual RPSF more stable methods
Yu et al. [2], 2015	Tile B1	A. Single 6.5 mm PCSF B. 3.5 mm superior RPSF	Axial load 600 N (single-leg standing)	Whole stress, displacement of the bilateral pelvis, stress analysis of implants	3D finite element model analysis	Both PCSF and RPSF biomechanically adequate
Yu et al. [30], 2015	Intact pelvis	Single 6.5 mm PCSF	Optimization of the secure trajectory of screws	Distance from surrounding major structures, screw trajectory parameters	A. 13 cadavers B. 3D finite element model analysis	Distance between round ligament of the uterus and pubic tubercle was 4.408 ± 0.304 mm, and between spermatic cord and pubic tubercle was 5.196 ± 0.251 mm. Study on parameters of screw channel in PCSF can improve the accuracy of the screw placement.

PCSF: percutaneous cannulated screw fixation; RPSF: reconstruction plate and screws fixation; M: males; F: females; AIIS: anterior inferior iliac spine.

**Table 3:** Quality assessment of the included biomechanical and anatomical studies.

Study	Objective Stated	Appropriate Study Design	Basic Information about Specimens	Conditions of Specimens	Study Protocol Clearly Stated	Exposure Accurately Measured	Outcome Accurately Measured	Results Presented Thoroughly	Stats Appropriate	Limitations Discussed	Clinical Implica-tions Discussed	Conclusions in Keeping with Results	Results Fit with Other Studies
Cano-Luis et al. [25], 2012	✓	✓	✓	✓	✓	✓	✓	✓	✓	✓	✓	✓	✓
Gonzálvez et al. [26], 2016	✓	✓	✓	✓	✓	✓	✓	✓	✓	✓	✓	✓	✓
Liu et al. [31], 2022	✓	✓	✓	✓	✓	✓	✓	✓	✓	✓	✓	✓	✓
O’Neill et al. [32], 2022	✓	✓	✓	✓	✓	✓	✓	✓	✓	✓	✓	✓	✓
Sun et al. [27], 2016	✓	✓	✓	✓	✓	✓	✓	✓	✓	✓	✓	✓	✓
Xu et al. [28], 2016	✓	✓	✗	✗	✓	✓	✓	✓	✓	✗	✓	✓	✓
Yao et al. [29], 2015	✓	✓	N/A	N/A	✓	✓	✓	✓	✗	✓	✓	✓	✓
Yu et al. [2], 2015 (1)	✓	✓	N/A	N/A	✓	✓	✓	✓	✗ *	✓	✓	✓	✓
Yu et al. [30], 2015 (2)	✓	✓	✓	✓	✓	✓	✓	✓	✗	✓	✓	✓	✓

N/A: Not applicable; *: Only for finite elements analysis.

**Table 4:** Complications reported in the included clinical studies.

Study	Technique	N	Implant Failure	Revision Surgery	Wound Infection
Chan et al. [24], 2022	PCSF	13	-	-	-
Chen et al. [22], 2012	A. PCSF	41	5	2	2
	B. RPSF	43	8	6	8
Eakin et al. [23], 2022	PCSF	12	-	-	-
Feng et al. [7], 2016	A. PCSF	16	3	1	-
	B. Tightrope and ex-fix	10	1	-	1
Mu et al. [3], 2016	PCSF	8	-	-	-
Yu et al. [2], 2015	A. PCSF	24	2	1	1
	B. RPSF	27	2	1	2

PCSF: percutaneous cannulated screw fixation; RPSF: reconstruction plate and screws fixation; N/R: not reported.

**Table 5:** Functional outcomes according to Majeed scoring system.

Study	Technique	N	Excellent (>85)	Good (70–84)	Fair (55–69)	Poor (<55)	*p*
Chan et al. [24], 2022	PCSF	12	2	2	4	4	-
Chen et al. [22], 2012	PCSF	41	23	12	5	1	0.01
	RPSF	43	10	24	5	3	
Feng et al. [7], 2016	PCSF	16	11	4	1	-	n.s.
	Tightrope and ex-fix	10	7	3	-	-	
Yu et al. [2], 2015	PCSF	24	18	5	1	-	n.s.
	RPSF	27	18	7	2	-	

PCSF: percutaneous cannulated screw fixation; RPSF: reconstruction plate and screws fixation; n.s.: non-significant.

## Data Availability

Not applicable.
